# Fabrication of Scaffolds for Bone-Tissue Regeneration

**DOI:** 10.3390/ma12040568

**Published:** 2019-02-14

**Authors:** Petra Chocholata, Vlastimil Kulda, Vaclav Babuska

**Affiliations:** Department of Medical Chemistry and Biochemistry, Faculty of Medicine in Pilsen, Charles University, Karlovarska 48, 301 66 Pilsen, Czech Republic; petra.chocholata@lfp.cuni.cz (P.C.); vlastimil.kulda@lfp.cuni.cz (V.K.)

**Keywords:** bone tissue engineering, scaffolds, regenerative medicine, stem cells, hydrogels

## Abstract

The present article describes the state of the art in the rapidly developing field of bone tissue engineering, where many disciplines, such as material science, mechanical engineering, clinical medicine and genetics, are interconnected. The main objective is to restore and improve the function of bone tissue by scaffolds, providing a suitable environment for tissue regeneration and repair. Strategies and materials used in oral regenerative therapies correspond to techniques generally used in bone tissue engineering. Researchers are focusing on developing and improving new materials to imitate the native biological neighborhood as authentically as possible. The most promising is a combination of cells and matrices (scaffolds) that can be fabricated from different kinds of materials. This review summarizes currently available materials and manufacturing technologies of scaffolds for bone-tissue regeneration.

## 1. Introduction

Tissue engineering is a relatively new and a very multidisciplinary field. It interconnects many disciplines, such as materials science, mechanical engineering, clinical medicine and genetics [1]. The main objective of tissue engineering is to restore and improve the function of the tissues by preparing porous three-dimensional scaffolds, and seeding them with cells and growth factors [2]. These three things (scaffolds, cells, growth factors) are known as “the tissue-engineering triad”, and this system is set up in an appropriate environment in a bioreactor [3,4]. The term “tissue engineering”, where engineering and the life sciences are interconnected, was introduced in 1988 in the National Science Foundation workshop as “the application of principles and methods of engineering and life sciences towards the fundamental understanding of structure–function relationships in normal and pathological mammalian tissues and the development of biological substitutes to restore, maintain or improve tissue function.” Langer and Vacanti used this term in a review article published in Science in 1993 [5].

Tissue engineering uses different techniques and different systems. The simplest approach is delivery of suitable signal molecules (tissue-inducing substances) to the right place. Most techniques rely on cells or cell substitutes, which can replace non-functional cells. The challenges are to preserve their function and immunological rejection. The most promising are combinations of both cells and matrices, as a matrix can be used in different kinds of materials as a matrix, and there is a tendency to investigate bioactive materials that mimic the native biological neighborhood [6]. This system could be implanted or used as an extra-corporeal mechanism, where cells placed on or within matrix systems can be open or closed (encapsulated). An open system is fully integrated into the body. The cells are anchored in a matrix and the system is implanted into the body. Use of immunosuppressive drugs or autologous cells is a way of avoiding immunological rejection. A closed system is protected from the patient’s immune system by a membrane that isolates the cells but allows nutrients and waste to permeate [5].

Strategies and materials used in oral regenerative therapies correspond to techniques generally used in bone tissue engineering.

## 2. Bone Tissue Engineering

Bone tissue engineering could be a way of repairing bone defects generated from various causes. This field comprises three main parts in vivo or in vitro—seeding cells, growth factors and scaffold materials [7].

The main target of tissue engineering is to simulate natural behavior. The big advantage of bone tissue is its natural self-repairing, remodeling and regeneration. There is an aim to produce scaffolds able to provide regenerative signals to cells. Scientists try to develop a way of producing scaffolds made of biomaterials that mimic those found in the natural environment, with multi-functional properties, such as improving cell adhesion, proliferation, and differentiation [8].

Bone transplantation is the second most common type of tissue transplantation following blood transfusion. Due to the aging of the population, it is expected to be increasingly in demand [9]. Bone tissue engineering combines biomaterials and cells. In the field of bone regeneration, emphasis has been placed on stem cells [10], particularly in connection with osteoblasts.

Based on principles of modern tissue engineering, craniofacial tissue engineering emphasizes craniomaxillofacial applications and aims to develop biomaterials for regeneration of oral and dental tissues, such as bone, dentin, cementum, periodontal ligaments, mucosa, and salivary glands [11]. Pulp cells were tested on a synthetic polymer scaffold in vivo and in vitro to regenerate the pulp, and it was the fundamental research for pulp-dentin tissue engineering [12].

Regenerative endodontics (RE), a new part of regenerative medicine, aims to treat infection of the dental pulp that can lead to inflammation and even to tissue necrosis. Root canal system treatment may be a common way, but it could lead to re-infection and even to tooth loss at the end. RE tries to discover alternative methods of pulp and dentin regeneration. Cell therapy is becoming a pivotal part of RE, and human permanent dental pulp tissue stem cells (DPSCs) could be a promising source of stem cells. DPSCs can be accessed easily, and they could be able to differentiate into various lineages (e.g., fibroblast, nerve cells, endothelial cells and odontoblasts) to create new connective tissue [13]. An easy source of DPSCs could be human third molars, due to their high level of clonogenicity and proliferation and ability to form calcified colonies [14]. The main target of RE is to develop suitable scaffolds, including antibiotic mixtures, cells, and growth factors, for pulp-dentin complex regeneration. Scaffolds could be produced from polymers, both synthetic (e.g., poly(lactic) acid) and natural (e.g., collagen) by various technologies [15].

Guided bone/tissue regeneration (GBR) is the most well-documented technique of periodontal regenerative therapy [16]. GBR, also called “membrane protected bone regeneration” [17], uses barrier membranes in the treatment of alveolar ridge defects and promotes bone growth into tissue defects adjacent to dental implants. Mineralization of the newly formed bone matrix at the GBR region could develop earlier than at the bone-implant interface, and it causes local mechanical stability due to regional variation of interfacial bone properties [16].

## 3. Structure and Properties of Bones

### 3.1. Architecture of Bones

**Figure 1:**
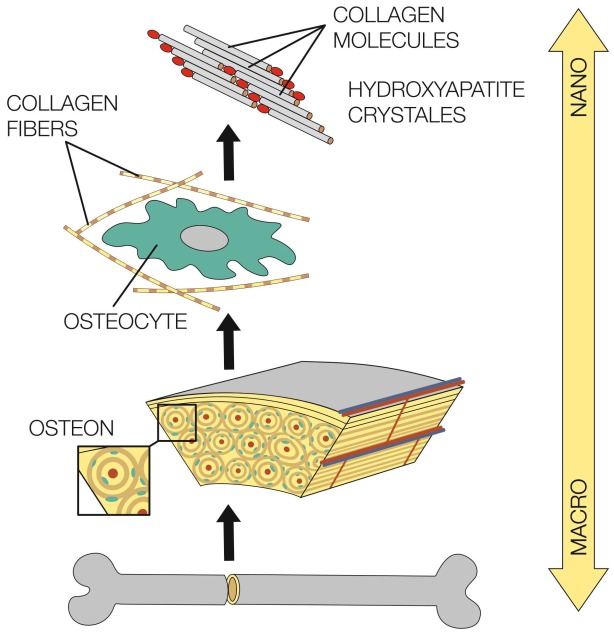
Different length scales in hierarchically organized bone. The macrostructure creates the overall bone shape and consists of trabecular (cancellous, spongy) bone, 50–90 vol % porosity and compact (cortical) bone, less than 10 vol % porosity [20]. The microstructure (of about 10–500 μm) consists of the Haversian system, osteons and single trabeculae). The sub-microstructure (of 1–10 μm) consists of lamellae. The nanostructure (a few hundred nanometers—1 μm) consists of fibrillary collagen and embedded minerals. The sub-nanostructure (below a few hundred nanometers of minerals) consists of collagen, non-collagenous organic proteins, and fundamental structural elements.

The bone tissue is well organized from macro- to nano-scale structures (Figure 1) [10]. The bone extracellular matrix (ECM) consists of organic components (22 wt %), inorganic crystalline mineral components (69 wt %) and water (9 wt %). Organic components consist of type I collagen, also type III and type IV collagen, and fibrin [18]. In addition, there are over 200 types of noncollagenous matrix proteins (glycoproteins, proteoglycans, sialoproteins, etc.) [9]. Inorganic crystalline mineral components are represented by hydroxyapatite and calcium phosphate. Bone tissue contains the largest amount of calcium in mammals and it can be treated as a ceramic-organic bio-nanocomposite complex [9]. Organic components ensure flexibility, whereas inorganic components ensure strength and toughness [18]. The mechanical, biological and chemical properties and functions of bones depend on the irregular but optimized structure, making bone material heterogeneous and anisotropic, as can be seen at different levels (Figure 1) [18,19].

Two major types of bone structure can be distinguished: trabecular and compact bone. Trabecular bone is formed by a porous trabecular network and bone marrow filling a large inner space. Compact bone is made from inorganic crystalline mineral with a very low number of osteocytes, blood vessels, etc. Both types of bones are reinforced by collagen fibers. Age, anatomical site and bone quality influence the mechanical properties, the most important of which are strength and elasticity. Porosity and architecture affect the properties of trabecular bone. Compact bone is more resistant to longitudinal stress than to radial, and to compression than to tension [18].

### 3.2. Osteoblasts, Osteocytes, Osteoclasts and Bone-Lining Cells

During life, two inseparable processes—bone resorbing by osteoclasts and bone formation by osteoblasts—happen alongside remodeling of the skeleton with optimal mechanical integrity. Without integrity, there can be bone loss, especially in the form of osteoporosis [21]. Bone modeling takes place during growing up, as well as in adulthood, in which it maintains bending resistance and function. A long-term process of bone remodeling replaces damaged bone with new bone and maintains functions. Modeling and remodeling maintain formed bone and participate in repair of bone fracture. It has been established that about 25% of trabecular and 3% of cortical bone are removed and replaced every year [22].

**Figure 2:**
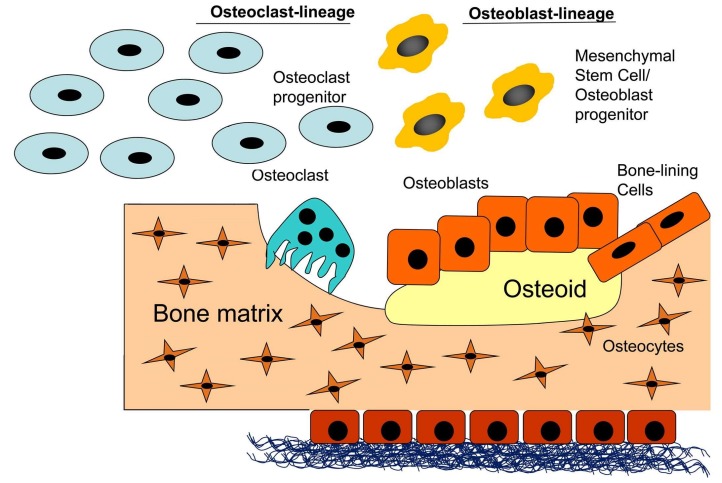
Matrix compartments of bone.

Marrow stroma is important for regulation of hematopoiesis. Endosteum, a source of mature osteoblasts in adulthood, comprising bone-lining cells, is important for the regulation of bone formation. Periosteum consists of two layers, a fibrous layer formed by collagenous tissue and a cambium layer containing a large number of cells. Cells in the cambium layer are activated during bone regeneration and fracture repair. Osteocytes (osteocyte perilacunar matrix) are present together with vasculature in a lacunocanalicular system (not mineralized), generating bone surface and participating in the production of ECM proteins that are important to phosphate metabolism and mineralization. An overview of bone ECM components can be seen in Figure 2 [21].

## 4. Materials

### 4.1. Bone Repair Biomaterials

Bone plays an important role in the homeostasis of minerals. The most important ones, phosphate and calcium ions, are stored there and, when required, they can be released into the blood. Another important function of bones is to ensure locomotion, load-bearing capacity and protection of the internal organs of the body. Bone tissue is dynamic and highly vascularized, a process that continues throughout an individual’s lifetime. Most fractures do not require any surgical intervention because of the high regenerative capacity of bone, but, unfortunately, large bone defects and non-union fractures do require it [20].

Treatment of degenerating tissues can be maintained by using an autograft or an allograft. Although both methods are revolutionary, there can be many problems that tissue engineering (regenerative medicine) needs to solve [3]. Autologous bone grafting, considered to be a gold standard in the treatment of bone defects, is a process in which a graft is taken from one anatomic site and implanted in another. Coming from the same individual, such a bone graft is integrated faster and more completely. On the other hand, there are several disadvantages, e.g., blood loss, longer surgical time, infection and a limited quantity of graft material [22]. The most commonly used autografts are cancellous, cortical, bone marrow and vascularized bone grafts [23,24]. During allogenic bone grafting, a graft is taken from one individual and transplanted to another. This type of graft can be adapted to an appropriate form. Allogenic bone grafting can be divided into cancellous, cortical and demineralized [22]. Bone grafts should satisfy the following requirements: osteoconductivity, osteoinductivity and osseointegration [25]. Osteoconductivity means that the bone could grow on the graft surface or down into pores, channels, or pipes, resulting in formation of cancellous bone in structure. In that case, mesenchymal stem cells can grow passively. Osteoinductivity is the ability to make pluripotent cells into bone-forming cell lineage. It is induced by growth factors that support mesenchymal stem cells in differentiating into chondroblasts and osteoblasts. During the osteogenesis, new bone is formed [23]. Osseointegration is related to direct contact between bone and the implant. Favorable incorporation of a graft is influenced by many factors, such as type of bone graft, site of implantation, etc. (Table 1) [24].

There is an effort to produce “bioactive” materials that can be integrated with biological molecules, in contrast to the past when “bio-inert” materials were designed [5]. Materials that can replace autologous or allogenic grafts consist of bioactive ceramics, bioactive glass, biological or synthetic polymers, and composites. With these materials it is easier to avoid problems with transplantation and implantation, such as infection or insufficient adaptation to environmental stresses. The ideal premise is that the material should be replaced by newly regenerated biological tissue at the same time [10].

### 4.2. Scaffolds

The term scaffold is used for three-dimensional (3D) biomaterial that provides a suitable environment for cells to regenerate tissues and organs. The aim is to produce scaffolds that are able to provide regenerative signals to the cells and to simulate natural behavior. Scientists try to develop ways of producing scaffolds comprising biomaterials that are very similar to the natural environment, with multi-functional properties [8], and which are efficient in terms of cost and clinical use [7].

The most important aspect is the structure of the scaffolds. Interconnected pores and high porosity allow cell attachment to facilitate 3D regeneration of tissue, cell growth, proliferation, and differentiation, diffusion of waste and the degradation products of scaffolds. The pore size must be large enough to allow migration of cells, but small enough to allow the binding of cells to the scaffold [7]. The degradation of the scaffold must last as long as the regeneration of the tissue [26].

Thus, an ideal scaffold for bone tissue would be osteoconductive, biodegradable and with proper mechanical properties. It is necessary to be able to deliver cells, to produce the scaffold in irregular shapes and, of course, to be commercially viable [27]. Tissue engineering aims to produce artificial constructs as 3D scaffolds containing appropriate cells implanted directly in vivo to stimulate and to direct formation of the new tissues. The human body is a sensitive system and materials for 3D scaffolds must be biocompatible, easily sterilizable, and have good mechanical properties [7].

Many types of scaffolds are made as hydrogels. A hydrogel is a 3D flexible network of natural or synthetic polymer that is insoluble in water [28], e.g., polyethylene glycol or alginate-based hydrogels [10]. It can retain a large amount of water or biological fluid due to hydrophilic groups such as –NH_2_, –COOH, –OH, –CONH_2_, –CONH–, or –SO_3_H. Hydrogels can mimic living tissues due to responding to changes in environmental conditions such as pH, temperature, and electric field. They are suitable for delivery in a largely non-invasive way and for in situ gelling at body temperature [29]. It can be applied in bone regeneration, and its big advantage is the possibility of cell encapsulation and chemical biofunctionalization [10].

Hydrogels are used as matrices for tissue engineering due to their porosity. Cells can grow and proliferate there, drugs can be released from them, nutrients and waste products can be diffused through them. Another benefit is easy modification by adhesion ligands. Self-cross-linking hydrogels, e.g., water-soluble chitosan and oxidized hyaluronic acid, are used for cell carriers because they do not need any chemical cross-linking agents [30]. On the other hand, there are also some disadvantages: due to their weak mechanical properties it is difficult to handle and sterilize them [31].

Classification of hydrogels can be based on different aspects (see Table 2) [28,29].

### 4.3. Types of Materials Used

From a historical point of view, the only important property of the first-generation biomaterials was their biocompatibility, while biointeractivity was the aim of the second-generation biomaterials. Whereas the first generation was passive, the second generation can rather promote tissue regeneration. Third-generation biomaterials are bioresponsive, e.g., they can activate genes to influence all aspects of proliferation and differentiation of cells [11,32].

Materials currently used for bone tissue scaffold fabrication are inorganic materials and natural or synthetic polymers. To enhance the mechanical properties of polymers, to utilize their excellent characteristics and to increase tissue interaction, composites of polymers and ceramics have been developed. It can be said that inorganic materials (bioactive glasses) are characterized by similar elastic modulus (about 40 GPa) as cortical bone (about 20 GPa). On the other hand, synthetic polymers feature lower values of strength (about 10 GPa) [33], and natural polymers even lower (about 70 MPa) [16]. Another important aim is to develop scaffolds with the ability to deliver specific drugs, such as growth factors or antibiotics, thus improving bone ingrowth, bone healing, and the treatment of tissue defects [34].

Natural materials, e.g., polysaccharides (starch, alginate, chitin/chitosan, hyaluronic acid derivates) or proteins (soy, collagen, fibrin gels, silk), help cell adhesion and function. However, immunogenicity may appear because of pathogenic impurities, and mechanical properties and biodegradability can be less easy to control [35].

Synthetic polymers, e.g., poly(lactic acid) (PLA), poly(glycolic acid) (PGA) and their copolymers, are more common in cell transplantation and scaffolds for tissue engineering because of their superior mechanical properties and degradation rate control [35].

Inorganic materials, e.g., metals, bioactive glasses, tricalcium phosphate (TCP), hydroxyapatite (HAp) and their combinations, are another group used in bone-tissue engineering because of their similarity to the bone mineral phase [10]. Other types of bone materials have been developed (e.g., HAp-TCP biphasic ceramics, wollastonite) [36,37]. The main advantages and disadvantages of different types of materials are summarized in Table 3.

#### 4.3.1. Metals

Metals might be considered the oldest material used for implants [38]. The first recorded use of metal implants was in Egyptian times [39]. The first metals used were aluminum, lead, gold and silver [40]. Nowadays, titanium and its alloys are the most frequently used metallic biomaterials for dental and orthopedic implants, as a result of their biocompatibility, non-toxicity and corrosion resistance. Commercially pure titanium has excellent biocompatibility but relatively poor strength. On the other hand, titanium alloys have superior strength, but contain ingredients that can be toxic or allergenic [41]. Metal alloys are applied as joint replacements and fracture-fixation implants, because of their good biocompatibility, corrosion resistance and strength [42,43]. Unfortunately, metals are not biodegradable, so there is usually a requirement to remove metallic implants, especially in the case of children [42].

#### 4.3.2. Bioceramics

Bioceramics include mechanically strong biomaterials, e.g., ceramic composites, amorphous glasses and crystalline ceramics [42]. Bioactive glasses (BGs) and glass ceramics are biomaterials used not only in bone-tissue engineering but also in orthopedics and dentistry [44]. The most commonly used bioceramics in bone-tissue engineering are HAp, TCP and their composites [42]. Ceramic scaffolds of HAp and TCP applied for bone regeneration are characterized by high mechanical stiffness, very low elasticity, and brittleness. Due to their chemical similarity to native bone they show excellent biocompatibility and facilitate good differentiation and proliferation of osteoblasts. On the other hand, their brittleness can cause problems with mechanical loading and degradation-rate control [1].

Bioactive glasses (calcium and phosphate containing silica glasses) produce bioactive hydroxyapatite after immersion in biological fluid and are able to bond to biological tissue. They can improve differentiation and osteogenesis because they deliver silica ions that are necessary for activating gene-transduction pathways [45]. It takes years to resorb bioactive glasses and bioceramics with crystalline HAp. For better reabsorption, other calcium phosphates can be used, but their disadvantages include low strength and brittleness, which are the characteristics that make it impossible to use inorganic materials for load-bearing applications [10]. The most usual applications of bioactive glasses are bone-filling materials, small bone implants, coating orthopedic implants and dental applications [44]. The two main manufacturing processes used to make bioactive glasses are melt-quenching and the sol-gel route. Before quenching oxides in water, they are melted at a high temperature [42]. Bioactive glasses and ceramics are able to form a layer of active hydroxy carbonate apatite (HCA), which connects to tissue upon implantation owing to their chemical and structural similarity to the bone mineral phase. Ceramics can be modified to create an apatite layer in vivo in protein-free simulated body fluids (SBF) [34].

Hydroxyapatite (HAp) (Ca_10_(PO_4_)_6_(OH)_2_), as a major natural inorganic component of bone, shows excellent bioactivity, biocompatibility, osteoconductivity, non-toxicity and non-inflammatory characteristics. Synthetic HAp is white, whereas natural HAp can have various colors (brown, yellow, green) [9]. Its mechanical properties are essentially influenced by the size of the HAp particles [46], porosity, density, etc. [9]. HAp is very hard but brittle, with a very slow degradation rate in vivo, and that is why it should be joined with natural or synthetic polymers to create scaffolds. On the other hand, HAp is very beneficial for constructing bones, because it stimulates growth factors (e.g., bone morphogenic protein) and encourages alkaline phosphatase (ALP) in mesenchymal stem cells (MSCs) [42].

Tricalcium phosphate (TCP) supports in vivo osteogenic differentiation of MSCs and is usually used for the production of scaffolds. Injectable 3D scaffolds of beta-TCP (β-TCP), in combination with alginate gel and also with type I collagen, are also available. Collagen scaffolds in combination with TCP implanted in a rabbit femur bone showed better bone formation than collagen-HAp scaffolds [42].

#### 4.3.3. Polymers

Two types of biodegradable polymers are used. The first are natural polymers and the second are synthetic. Natural polymers such as polysaccharides (starch, alginate, chitin/chitosan, hyaluronic acid derivatives) or proteins (soy, collagen, fibrin gels, silk) provide admirable cell attachment and growth. On the other hand, they have many disadvantages, e.g., immune-response problems, and poor mechanical properties [10]. The challenge in this field of material engineering is to produce natural polymer-based scaffolds of sufficient quality and homogeneity [3].

Collagen is a basic component of animal tissues, such as bone, cartilage, tendons, skin, and blood vessels [3]. Its polypeptide chain is very flexible and contains mostly glycine, proline, hydroxyproline and lysine. The degree of flexibility is determined by the amount of glycine. Although about twenty-nine types of collagen are known, the most widespread is type I [42]. As collagen is the main part of ECM, it fulfils all the requirements for biomedical applications. Its principal advantage is enzymatic biodegradability [47]. The process of biodegradability, as well as mechanical properties, can be modified by cross-linking or by combination with inorganic compounds, e.g., HAp [42]. Collagen-HAp scaffolds have been produced with optimal pore structure for bone regeneration [3], where 99% interconnectivity and excellent cell infiltration have been achieved. Scaffold derived from mouse-bone marrow from mesenchymal stem cells was implanted into a mouse calvarial defect. After three weeks, the defect was annealed and after several weeks, degradation of collagen-HAp the scaffold was proven successful. Furthermore, it was confirmed that the osteogenic differentiation of cells was improved by the addition of magnesium nanocrystals. Using human-derived bone MSCs with this type of scaffold could be a way of treating osteochondral defects [42].

Gelatin is derived from collagen [48] by breaking the structure of the triple-helix into a single strand [49]. This material is biocompatible as well as biodegradable because of its amino acids (such as arginine-glycine-aspartic acid), which also encourage cell adhesion, migration, differentiation and proliferation [48]. Although gelatin comes from collagen, its antigenicity is lower, but there are still some information signals [50]. Change of its solution temperature causes gel formation, which is utilized not only for wound dressing [51], but also for delivering a variety of drugs. The poor mechanical properties of gelatin gels have led to the production of gelatin-based composite scaffolds (e.g., ceramic-gelatin) for cartilage and bone repair [47].

Chitosan is one of the most widely used materials for scaffolds. In bone-tissue engineering, it can be used alone or with other polymers or ceramics [52]. Chitosan has the same structure as a non-collagen organic component of ECM glycosaminoglycan (GAG) [48]. This is a linear polysaccharide consisting of *D*-glucosamine and *N*-acetyl-*D*-glucosamine linked by β (1-4) glycosidic bond [47,53]. Chitosan can be obtained by deacetylation of chitin, which is an exoskeleton component of crustaceans [54]. Free amine groups facilitate inherence through a positive charge for binding with different negatively charged molecules (e.g., lipids, cholesterol, metal ions, proteins, etc.) [49]. The degree of deacetylation affects the crystallinity of chitosan, and 100% deacetylated chitosan is highly crystalline [55]. Chitosan might support cell attachment, differentiation, and migration [48], and because of its non-toxicity, non-allergenicity, mucoadhesivity, biocompatibility, biodegradability and osteoconductivity, it can be applied as a dental, bone or cartilage implant, or as artificial skin. Cross-linked chitosan can be used as a bandage. Chitosan nanofibers used for burn healing can protect against infection, absorb exudate, provide air access to a wound and help in the regeneration of skin tissue [47]. It has been stated that chitosan promotes the growth of osteoblasts and the mineralization of matrices [56], and for that reason it is extensively used as a sponge in bone-tissue engineering. Chitosan sponge is a flexible, soft material with interconnected pores but with poor mechanical strength [47] and therefore it is very often mixed with other natural polymers or bioceramics to obtain scaffolds. 3D scaffolds of HAp/chitosan-gelatin present a structure that is similar to human bone. The presence of HAp improves mechanical properties, and the combination of natural polymers, ceramic material and cells exhibits the effect of biomineralization after three weeks [57]. Chitosan-based composite biomaterials are not only a very good option for cartilages and intervertebral discs, but also for gene therapy in orthopedics.

Hyaluronic acid (HA) is abundant throughout the extracellular matrix of the human body. It is composed of a linear glucosaminoglycan, where units of *N*-acetyl-*D*-glucosamine and *D*-glucuronic acid are repeated [58]. Not only its biocompatibility and biodegradability but also its viscoelasticity are convenient properties for using HA in biomedicine [47]. A very significant advantage of HA is its enzymatic degradability by hyaluronidase [59], an enzyme produced by mammalian cells [47]. In view of the very rapid degradation and water solubility of HA, it is advisable to cross-link it. HA hydrogels can be produced through its chemical modification by mono- and polyvalent hydrazines [60], and HA in this form is used for scaffolds. Modified HA can be used in the form of sponges, obtained by lyophilization, for the culturing of human fibroblasts, chondrocytes and bone marrow MSCs [61]. Excellent adhesion and proliferation of human adipose-derived MScs on heparin-HA hydrogel) was found. The heparin significantly increased cell adhesion compared to HA alone. When ADSCs were encapsulated into heparin-HA hydrogel, a distinctive expression of hyaluronidase was observed. Heparin-HA hydrogels with encapsulated stem cells without any modification could be a promising system for application in biomedicine [59].

Alginate is a polysaccharide composed of β-*D*-mannuronic acid and α-*L*-guluronic acid connected by (1-4) bond [47]. Its biocompatibility and ability to gel easily facilitate alginate which can be used in many biomedicine applications [62]. Anionic polymer alginate can be obtained from seaweeds and is already in commercial production [47,62]. Commercially used alginate presents higher purity and therefore there is hardly any inflammatory response [62]. Alginate can be easily modified chemically, and use is often made of this in its applications, e.g., wound-dressing materials, drug-delivery systems, and implantation of protected living cells [63]. Tissue engineering uses alginate as a delivery system via encapsulation techniques. The first encapsulation was used for rats’ Langerhans islets to transplant cells to affect type I diabetes. Application in mammalian reproductive technologies was started by encapsulation of bovine sperm [47]. In mammals, alginate is non-degradable because of lack of enzymes, but degradability can be increased by its ionic cross-linking [62], and the degradation can proceed not only in an acid (pH < 5.0) or basic (pH > 10.0) environment, but also in a neutral environment in the presence of reducing compounds. Unfortunately, sterilization, e.g., by heating, autoclaving, ethylene oxide or γ-radiation, causes degradation of alginate [63]. Alginate gels are widely used in bone-tissue engineering for bone and even cartilage regeneration because gels can be easily introduced into the body in a minimally invasive way and irregular shapes can be filled in.

Agarose is another polysaccharide that is useful for tissue engineering [64]. It consists of repeating units of agarobiose (1,3-linked disaccharide of β-*D*-galactose and 3,6-anhydro-α-*L*-galactopyranose) [65]. Agarose is often combined with other polysaccharides to form hydrogel scaffolds [64] because of its poor cell attachment [65]. Agarose is a widely used compound not only in tissue engineering and drug controlled release, but also as a gel for electrophoresis, chromatography and, due to agar similarity, as a culture medium [66]. It can be obtained by extraction of agarophyte seaweed (Rhodophyceae algae) cell walls [64]. Agarose is soluble in hot water and can be prepared in the form of a thermal-reversible gel. Its temperatures for gelling and melting are from 30–40 °C to 80–90 °C, depending on molecular weight, concentration and the number of its side groups [65]. The mechanism of agarose gelation depends on hydrogen bonds; helical structure is formed at first and then the gel appears [67]. There is no need for cross-linking agents, because of the formation of hydrogen bonds [65]. Due to the fact that mechanical properties can be adjusted, agarose can be available for different applications in tissue engineering, especially in bone and cartilage scaffolds. Agarose-based materials can create 3D hydrogel scaffolds that provide an appropriate environment for cell growth including good permeability for oxygen and nutrients. By adding HA into agarose composite, agarose/HA hydrogels were created and pore size, swelling ratio and thermal stability increased. Little inflammatory response in vivo was reported. The HA content determines the rate of degradation: the higher the HA content, the higher the rate of degradation [68]. Composites containing HAp showed higher ALP activity, and 3% of micro-HAp was confirmed as the optimal amount for calcification [69]. New Zealand rabbits were used for an animal experiment. HAp/agarose gel composite was injected in the drilled holes, and the observation periods were 1, 2, 4 and 8 weeks. Pure agarose gel was used for the control group. After 8 weeks, excellent bone formation was observed by using micro-computed tomography analysis. After 4 weeks, little bone regeneration was observed in bone defects containing pure agarose gel [70]. There is a need for more systematic research involving agarose-based biomaterials, but these biomaterials will definitely find clinical applications [65].

Synthetic polymers are based on polyesters, such as polylactic acid (PLA), polyglycolic acid (PGA), poly ε-caprolactone (PCL) or poly (lactic-co-glycolide) (PLGA) copolymers [47]. They can be produced with a tailored structure, and degradation rate can be controlled quite easily [3]. Their disadvantage is that there can be problems with their reduced bioactivity.

Poly(α-hydroxy acids) including poly *L*-lactic acid (PLLA), polyglycolic acid (PGA) and poly *D*,*L*-lactic-co-glycolic acid (PLGA) copolymers, are the most widely used synthetic polymers for 3D scaffolds in bone-tissue engineering, but were historically used as resorbable surgical sutures. According to need, the degradation rate can be adjusted from weeks to several years. The degradation products can be excluded from the body as carbon dioxide and water [3]. To support cell distribution and diffusion of nutrients, meshes, fibers, sponges and foams are produced as scaffold types. There are several disadvantages to these materials. The first one is that these polymers degrade by bulk erosion, which causes premature degradation [47]. Increasing amounts of acidic degradation decrease pH, which can accelerate the degradation rate and eventually cause inflammation. Another disadvantage of porous scaffolds is their relatively poor mechanical properties, especially in vivo [64]. It is possible to obviate these disadvantageous properties by preparing copolymers, composites of polymer/bioactive ceramic [47], especially HAp, for bone-tissue engineering [64], or by adjustment of molecular weight [47]. It has been reported that compact PLLA/HAp composites have good osteoconductive properties, a better cell environment for seeding and growing [71], and surface characteristics that are important for osteoblastic cells [47]. Degradation products do not change pH, which was detected during 24 weeks of monitoring, and acidic products might act as buffers. Not least, the mechanical properties were also improved [71]. 

Seeding and delivering the cells into porous scaffolds of PLA and PLGA is negatively affected by the hydrophobicity of the polymer surface, which is another reason for combination with HAp and bioactive glasses [47]. Porous polymers with a pore size of 100–500 μm, combined with bioceramic particles, were found to be optimal scaffolds for bone-tissue engineering. The degradation rate, as well as potential inflammation, can be affected by the composition of the polymers. Because of possible toxicity of the residual solvent, it was found that the optimal production techniques were the solvent-free method, gas foaming and rapid-prototyping [72].

Poly(ε-caprolactone) (PCL), a semi-crystalline, biodegradable [73], non-toxic in nature [74], aliphatic polyester, with a low melting point (60 °C) [47], can be used for easy production of scaffolds for tissue engineering [75], bone and cartilage repair, surgical sutures, and drug-delivery systems [47]. Although it is more stable, cheaper and readily available, and in higher quality, than polyhydroxy acids [76], the main disadvantages of PCL are its hydrophobicity, which is disfavorable for cell attachment and infiltration, and its slow degradation, which can last up to 3 or 4 years [75]. Modification of its properties can be achieved by co-polymerization or blending with other polymers. Co-polymerization directly changes chemical properties that influence other properties indirectly. In comparison with blending, it changes physical as well as chemical properties and the biodegradation rate [77]. Blending PCL with bioactive glass and bioceramics, such as HAp, can improve hydrophilicity and bioactivity of scaffolds. It was found that a blend of PCL and magnesium phosphate (MP) contained interconnected pores and achieved porosity of 73%. MP particles increased hydrophilicity and the MP can be used for controlling the degradation rate [75]. Co-polymerization of ε-caprolactone with methoxy poly(ethylene glycol) block copolymers for drug-delivery systems can be obtained and hydrophilicity and even lipophilicity can be altered [73]. PCL can be combined with both natural and synthetic polymers [76]. Scaffolds of PCL prepared by solid free-form fabrication (SFF) can differ in internal architecture and porosity. Even the selective laser-sintering method (SLS) seems to be sufficient for PCL scaffolds used in bone and cartilage tissue engineering. The mechanical properties of such PCL scaffolds are within the lower range of trabecular bone. Scaffolds can sustain appropriate stress, and SLS is an easy method for scaffold production [76].

Polyurethanes (PU), a major class of synthetic elastomers [78], are products of reaction of molecules with two or more hydroxyl groups and molecules containing two or more isocyanate groups. Two thermodynamic incompatible phases can be obtained [77]. Polyester chains comprise the soft segments, while the hard segments are composed of polyurethane blocks on aromatic isocyanates, providing non-biocompatibility due to the toxic degradation products. If polyurethanes are designed to have chemical linkages, they can be degraded in the biological environment. The toxicity of degradation products, the main disadvantage of polyurethanes, could be reduced by using lysine diisocyanate (LDI) or other aliphatic diisocyanate (e.g., hexamethyl diisocyanate, 1,4-butandiisocyanate). Another way to prepare biodegradable and non-toxic polyurethanes can be using star-shaped polyester prepolymer (from myoinositol). This type of the polyurethane was implanted in guinea pig, where the biodegradability was demonstrated [78]. Polyurethanes are used for fabrication of medical implants, especially for long-term implants [70] and biomedical products such as cardiovascular catheters, diaphragms of blood pumps, coating materials for implantable pacemakers, etc. [78].

#### 4.3.4. Composite Materials

As we have seen above, there are many problems with scaffolds produced from a single biomaterial (advantages and disadvantages listed in Table 3). The best solutions at present are composite scaffolds, i.e., a combination of ceramics and polymer, or of synthetic polymers with natural polymers [1]. Composite materials include a polymer phase with toughness and compressive strength and an inorganic phase with bioactivity, which improves the mechanical properties and degradation rate. That is why these materials are very similar to the natural structure of real bone. Sol-gel processing is a technique that can create a polymeric network with inorganic components. Unfortunately, their mechanical properties are not yet as good as the mechanical properties of bone [10]. Each phase has different properties, and tissue-engineering templates must interconnect the best properties of both kinds [79].

Composite metal scaffolds can achieve an increase in biodegradability [80]. Magnesium (Mg) is one of the most promising metals in combination with nutrient elements, strontium (Sr), calcium (Ca), and alloys of Mg–Sr, Mg–Ca–Sr were produced and evaluated in vitro with bone marrow-derived MSCs. The study showed that ternary Mg–Ca–Sr alloys had a higher degradation rate and better cell adhesion compared to binary Mg–Sr alloys. Generally, Mg–1Sr, Mg–1Ca–0.5Sr and Mg–1Ca–1Sr were recommended as the most suitable for in vivo studies in animals and also for clinical testing [80]. Another success of proliferation and osteogenic differentiation was achieved by preparing composites of Sr, HAp and chitosan with human bone marrow MSCs. Improvement of attachment and proliferation was also achieved for composites of nickel-titanium treated with sodium hydroxide, titanium-based scaffolds, porous 3D injected iron-magnesium scaffolds, composite titanium-silica scaffolds with complex geometry and stainless steel, titanium and cobalt chromium alloys [42]. Porous Nb–Ti–Ta alloys can induce apatite in vitro and that is why they might be a new candidate for application in bone-tissue engineering [43]. Many bio-inorganic ions (silicon, zinc, copper, lithium and cobalt) incorporated into graft material for bone-tissue engineering proved equally beneficial for healing bone defects, but further research is needed [22].

## 5. Manufacturing Technology of Composite Scaffolds

### 5.1. Methods of Scaffold Fabrication

Incorporation of advantageous properties allows the production of composite scaffolds [34]. There are several processing technologies that have been shown to produce porous 3D polymeric scaffolds for bone tissue, especially used is solvent casting and particulate leaching, gas foaming, emulsion freeze-drying, electrospinning, rapid prototyping and thermally induced phase separation [81].

#### 5.1.1. Solvent Casting and Particulate Leaching

The solvent casting and particulate leaching technique is the most common and easy method, where pore size and porosity (depending on the salt/polymer ratio) can be controlled. This technique consists of dissolving polymer in an organic solvent, mixing it with water-soluble porogen, such as salt (e.g., sodium chloride, sodium citrate) [82], and casting the resulting mixture into a mold [34]. The solvent evaporates or lyophilizates, and the polymer/porogen composite is leached into water. The porosity of the scaffold is dependent on the amount of porogen, and the size of the pores depends on crystal size. Waxy hydrocarbons [82] and gelatin particles [83] are alternative porogens. It was found that a high interconnectivity of pores could be achieved at 70 wt % of porogen. The solvent-casting method does not require any special equipment (flat sheets and tubes) [82]. Remaining toxic solvent can cause denaturation of incorporated molecules, a decrease in the activity of bioinductive molecules [34], and impossibility of adding pharmacological agents [82]. The polymer-ceramic 3D scaffolds obtained feature-controlled pore interconnectivity and porosity at low porogen levels [34].

#### 5.1.2. Gas-Foaming Process

The gas-foaming process is a technique where it is possible to avoid organic solvents, and pores are created by gas expansion [84]. Carbon dioxide is used as a porogen gas [82], and this common gas is low-toxic and non-flammable [84]. This method affords highly porous foam (pore size of 100 µm) with a porosity of up to 93%, but the pore interconnection, especially on the surface, is low, at just 10%–30% [81]. Open porous scaffolds can be produced by using the gas foaming/salt leaching method. A combination of ammonium bicarbonate salt and acid substances at increased temperatures causes gaseous ammonia and carbon dioxide to be released. Macroporous structures with interconnected pores of 100–500 μm can be obtained where cells can be seeded and their viability is high. This is a simple and financially viable method of scaffold production [85]. This technique cannot be used for hydrophilic and glassy polymers, owing to low solubility in CO_2_, e.g., chitosan, but use of a co-solvent such as ethanol or diluted acid could solve the problem [84].

#### 5.1.3. Thermally Induced Phase Separation

Thermally induced phase separation (TIPS) allows production of porous anisotropic polymer scaffolds that can be easily controlled and which have a low probability of defects for tissues such as nerves, muscles, tendons, ligaments, the intestines, bones and teeth [86]. Their properties, e.g., pore morphology, mechanics, bioactivity and degradation rate, depend on polymer concentration and the volume of the secondary phase fraction [81]. Generally, a polymer is dissolved in a solvent at a high temperature, and a porous polymer scaffold is obtained by cooling the homogenous solution, which causes phase separation, and a microporous structure arises after removal of the solvent [86]. Because of pore size (10–100 µm), however, this is not very suitable for seeding of the osteoblasts or for bone-tissue growth. Therefore, a coarsening process is needed in the thermally induced phase in order to generate a pore size greater than 100 µm. The properties of scaffolds depend on polymer concentration, solvent type and temperature gradient. The use of organic solvents is a disadvantage of this technique, because solvent sublimation takes a long time [81].

Dimethyl sulfone (DMSO_2_) was recommended as a universal crystallizable solvent for polar polymers. As a result of the use of DMSO_2_, TIPS can be called a green method, because the solvent can be recovered by recrystallization and sublimation [86]. Another type of solvent is PolarClean^®^ (sonicated Methyl-5-(diethylamino)-2-methyl-5-oxopentanoate), which is described as environmentally friendly because it is non-toxic, water-soluble and biodegradable [87].

#### 5.1.4. Solid Free-Form Fabrication Technique

The solid free-form fabrication technique (SFFT), also known as rapid prototyping (RP) or additive manufacturing, belongs to the computer-controlled methods, such as shape- or size-solving for different organs and tissues [88,89]. Use is made of a computer-aided design (CAD) model [81], where the patient’s specificity is harmonized with the structural requirements of the scaffold, which results in a complex, highly accurate, 3D product [88,89].

The process comprises several steps. The first step is to create a CAD model which is transferred to a file suitable for stereolithography method. In the “pre-processing” step, there is a stereolithography file digitally sliced into cross-sectional layers. Then one layer is produced, and the printing continues until the process is completed. The finishing of the structure involves hardening and surface treatment [88]. Digital data, which facilitate production of precise structures [34] of porous (over 90%) and interconnected scaffolds [89,90], with high reproducibility [88], are received from imaging techniques such as computer tomography or magnetic resonance. SFFT provides scaffolds with controlled micro- and macroporous structures [34] in different parts of the same scaffold. Anisotropic microstructures can be beneficial where multiple cell types are essential. Different SFFT used by different research groups are evaluated in Table 4 in respect of the advantages and disadvantages of their technique types. Solid free-form fabrication is a relatively new method. The solution can be found very quickly, and the scaffold is tailored. Not all SFFT types are used for production of scaffolds [88,89,90,91].

#### 5.1.5. Microsphere Sintering

In this method, a microsphere composite of ceramic and polymer that is produced by the emulsion/solvent evaporation technique, is sintered and 3D porous scaffolds are obtained. The porosity obtained is about 40% and pore diameter is about 90 μm [34]. Sintered microsphere scaffolds have excellent mechanical properties comparable to cancellous bone. Sintering temperature and sintering time are the decisive factors. Higher temperature and a longer time give superior fusion of microspheres, a smaller pore size with lower porosity, and better mechanical properties [92].

Using a solvent (most frequently methylene chloride and acetone) can be another way of producing sintered microsphere scaffolds [92]. PLGA, as the most often used synthetic polymer, was used for microsphere sintered scaffold with two types of pores. Larger pores enable blood vessel and bone tissue ingrowth and smaller surface ones improve the passage of nutrients. Thanks to smaller surface pores, surface roughness is increased, and cellular attachment and proliferation are improved [93].

CO_2_ was used for a subcritical CO_2_ sintering method. The gas-foaming method uses CO_2_ currently, but this method creates a closed-pore structure. It has been claimed that the use of CO_2_ for producing scaffolds with microsphere sintering creates interconnective pores with higher porosity. The optimal range of CO_2_ pressure was found to be between 15 and 25 bar. The advantage of using CO_2_ is its non-toxicity. More research is needed into establishing and setting sintering conditions with CO_2_ and adding growth factors without damage occurring [94].

#### 5.1.6. Emulsion Freeze-Drying Method

The emulsion freeze-drying method is based on phase separation including emulsification and freeze-drying [82], and highly porous scaffolds are produced. The first step is preparation of the emulsion by homogenization of a polymer in an organic solvent and water. This emulsion is rapidly cooled down and liquid phases (water and solvent) are removed by freeze-drying. The resultant pores are close together, but the porosity is higher than 90% and the size of pores is between 20 and 200 µm [81]. The emulsion freeze-drying method could be combined with particulate leaching, sucrose or sodium chloride can be added in to the emulsion to create porosity. After freeze-drying, particles can be washed away [95].

#### 5.1.7. Electrospinning Techniques

Electrospinning is a promising versatile technique that uses a high electric field to produce submicrometer fibers or nanofibers by reduction of surface tension within the polymer fluids. A solution or melt of a synthetic or natural polymer [96] is injected with an electrical potential to create a charge imbalance [81], which allows stable, steady deposition of electrospun fibers on any substrate. Different types of biopolymers (e.g., PCL, PU, collagen) could be electrospun [97]. Due to the poor stability of natural polymers and the harmfulness of the degradation products of synthetic polymers, a combination is usually utilized [96].

Electrospinning could generate non-woven matrices with nanoscale features. The thickness of individual fibers and their orientation could be controlled by type and concentration of the polymer and by the setting of electrospinning device. The polymeric non-woven nanofiber scaffolds have high porosity and high surface area [98].

#### 5.1.8. Three-Dimensional Bioprinting

Tissue engineering increasingly exploits 3D printing processes. 3D porous scaffolds need a consistent and adequate size of well-interconnected pores for cell migration and proliferation. Several commonly used techniques to produce these 3D scaffolds have been mentioned above, but all have the same disadvantages: inadequate control of scaffold architecture, pore network and size, and suboptimal 3D scaffolds [99]. These methods are not versatile enough in their processes [100]. 3D-printing methods could help to solve these drawbacks through rapid prototyping, solid free-form fabrication, biofabrication, bioprinting and additive manufacturing [101].

The basis of bioprinting is the creation of a defined structure in which cells are located by 3D bioprinter technologies [101]. More than 40 different 3D-printing methods have been developed, the most popular being fused-deposition modeling (FDM), stereolithography, inkjet printing, selective laser sintering (SLS), and color jet printing, which can process plastics [99]. 3D printing, as an additive manufacturing technology, connects computer-aided design (CAD), computer-aided manufacturing, numerical control techniques, laser techniques, polymers, and 3D computer tomography techniques [102], such as magnetic resonance imaging (MRI), computer tomography (CT), scanning, etc. A delivery medium is required for cells during the printing process in order to create the required shape from CAD generated by 3D medical images. The material for bioprinting is named bio ink, i.e., biomaterial-filled with cells. The huge advantage of 3D printing is that it does not require any physical masks or molds and it can transfer microscale and nanoscale into 3D structures relatively cheaply, flexibly and with high efficiency. All models are bespoke and very specific [103]. 3D bioprinting provides bio ink comprising well-structured biomaterials and/or living cells [100]. The three most common 3D printing technologies are: inject bioprinting/droplet bioprinting, extrusion-based bioprinting, and light-assisted bioprinting [104]. The most common materials for 3D bioprinted scaffolds are hydrogels or similar oxogenous materials. Unfortunately, only a few hydrogels are bioprintable [105]. Cells can be printed even without any scaffold, by imitating embryonic development. First of all, neo-tissue is formed from cells, deposited into specific patterns for fusing and maturing, and then larger-scale functional tissue is obtained [101]. Unlike conventional 3D printing, bioprinting requires different techniques, facilitates living cells, and assures control of cell distribution and deposition. It is also relatively cheap [105].

3D bioprinting can be considered as a new and very promising approach to producing 3D tissue structures, but it is necessary to evaluate different bio-ink materials in order to satisfy property criteria [100]. Other targets are to minimize loss of cells, to intensify cell interactions and to vascularize tissue structure [101]. Despite this, it is already possible to create cell-laden perfusable vascular constructs by 3D bioprinting, which will be applied in engineering prevascularized tissue constructs [106].

The main problem of bone-tissue engineering, or generally of tissue engineering, is vascularization of tissue. Zhu et al. attempted to study the architecture of the vasculature network and developed a new level of accessibility based on rapid 3D printing: microscale continuous optical bioprinting (μCOB) for vascularized tissue with derived biomaterials. Two types of biocompatible and photopolymerizable hydrogels—glycidal methacrylate-HAp, gelatin methacrylate and human umbilical vein endothelial cells from passage 3–6—were used for the bioprinting. Vascularized 3D tissue can be created by microscale continuous optical bioprinting together with a prevascularization technique using other primary or stem cells [107].

3D printing, as an additive manufacturing technology, is certainly a part of tissue engineering, but it will take a long time to transform academic products into clinical ones. Currently, the main target is to standardize and certify 3D-printed medical devices and to solve legal problems [99].

Electrohydrodynamic-jetting (EHD-jet) technology is a novel very versatile fabrication method facilitating generation of micron- to nanoscale fibers and even micron- to nanoscale scaffolds [108]. Orientation of fibers can be controlled precisely by computer. Fabricated scaffolds can have high-resolution patterns, accuracy, well-controlled structure, and optimal pore size and porosity. Briefly, a layer-by-layer process uses a high voltage between the nozzle and substrate, and a very thin fiber is extruded. EHD jetting is similar to electrospinning but the main difference, in terms of the process parameters, is applied voltage and the distance between the nozzle and the substrate. While electrospinning usually uses a voltage higher than 10 kV, EHD jetting uses a voltage of only between 2 and 3 kV. The distance between the nozzle and the substrate is higher than 5 cm in electrospinning and less than 4 mm in EHD jetting [108,109].

The manufacturing technologies of scaffolds are summarized in Table 5.

#### 5.1.9. Bioreactor

A bone is a dynamic system; therefore, culturing of vascular tissue should also take place under dynamic conditions, to ensure maximal perfusion of nutrients and oxygen [110]. The main aim of bone-tissue engineering is to ensure sufficient supply of nutrients and oxygen into the inner part of scaffolds. It was demonstrated that improving nutrient supply and intrinsic shear stresses by medium flow significantly influences the expression of osteogenic genes in osteoblasts. A bioreactor facilitates direct perfusion of the culture medium [111], and thereby physiological conditions are imitated during cultivation [112]. In broad terms, a bioreactor is a system where it is possible to control and monitor conditions [113]. In bone-tissue engineering, bioreactors are used as in vitro tools to imitate the natural environment in the generation and growth of new tissue. Controlled conditions (pH, temperature, oxygen tension, perfusion of the cells, mechanical forces) can be modified as required. Bioreactors must be designed to satisfy several requirements: they must be easily deposable, form new tissue in a short time period and keep everything sterile. Bioreactors must also be made of non-toxic materials, in respect of cells, and it must be possible to sterilize them [114].

The flow into scaffold pores is very important for osteogenic differentiation [115]. In bioreactors for bone formation, perfusion and shear stress should be ensured. Several dynamic methods were tested to ensure diffusion: cultivation on an orbital shaker, rotating-wall vessel bioreactors, spinner flasks, and a perfusion bioreactor. Spinner-flask cultivation increases cell osteogenic differentiation and even mineralization when compared with static cultivation. Shear stress generates a vortex field and improves mass transport into the scaffold [116]. Spinner-flask bioreactors are considered to be relatively inexpensive and simple [117]. To compare different flow rates during perfusion culture, as a mitochondrial activity of the cells, it can be stated that mitochondrial activity is higher at 0.1 mL/min flow rate than at 0.5 mL/min [115].

Rotating-wall vessel bioreactors are also used in bone-tissue engineering. It has been demonstrated that culturing rat osteoblasts in rotating-wall vessels increases expression and mineralization ten times more than culturing in shear flasks. This type of bioreactor facilitates better control of oxygen supply and less turbulence [114]. Scaffolds are placed between two cylinders, the inner one remaining stationary and the outer one moving. There is a danger of the scaffold free-falling under the influence of gravity, but this should be counteracted by the centrifugal forces [118].

A perfusion bioreactor is also used in bone-tissue engineering. The medium is directly pumped through the scaffold, and the exchange of nutrients and oxygen is ensured [114], as is a uniform distribution of cells [119]. This type of bioreactor is useful for large scaffolds. It was reported that the increase in flow rate also increased the amount of calcium. It can be said that perfusion bioreactors also encourage osteogenic differentiation and bone formation [114]. Some commercially developed bioreactor systems are available on the market; not all of them are for bone-tissue engineering, but it is possible to adapt ones that are not. Some of the most common commercial bioreactor systems are shown in Table 6 [120]:

Cells are very sensitive to their environment and to integration of biological components contained in the extracellular matrix, e.g., glycosaminoglycans or heparin tend to increase not only cell attachment, cell proliferation and osteogenic cell differentiation, but probably also scaffolds in an in vivo environment. To generate new tissue and to induce natural healing in bodies in bone engineering, it is necessary to create a controlled in vivo bioreactor environment. Such bioreactors are created between the tibia and the periosteum, mesenchymal tissue prolific in pluripotent cells. Gel rich in Ca supports bone ingrowth and generates bone that is very similar to native bone in its properties [10].

## 6. Cells Used for Bone Tissue Engineering

Adipose-derived stem/stromal cells (ADSCs), as well as mesenchymal stem cells (MSCs), can be used in tissue engineering and regenerative medicine because of their ability to differentiate into several lineages, their immune privilege, and their genetic stability in long-term cultures [121].

### 6.1. Human Adipose-Derived Stem Cells

It is generally known that it is possible to harvest ADSCs from adipose tissue, using a minimally invasive technique. ADSCs possess high plasticity, immunomodularity and angiogeneticity. According to the International Fat Applied Technology Society, human ADSCs are multipotent, plastic-adherent cells that are isolated from adipose tissue [121,122]. They seem very promising for cell-based therapy for many human diseases because of their biosafety and free immunogenicity. These cells are easily available in adequate amounts, and they can be passaged very easily and reliably [123]. It seems that stem cells of approximately 0.5 × 10^4^ to 2.0 × 10^5^ can be obtained from 1 gram of fat tissue, according to gender, age, body mass index and location of the fat tissue. In addition, there is no ethical problem, as there is for embryonal stem cells [124]. Their usage can be very wide because of their potential for differentiation [125] into many types of mesenchymal cells (osteoblasts, chondrocytes and adipocytes) with an appropriate expression of the major marker genes [124]. Using human ADSCs together with biocompatible material facilitates growing cells in 3D structures [123].

### 6.2. Human Mesenchymal Stem Cells

Scientists have been increasingly interested in the significant potential for use of MSCs [124]. Multipotent MSCs can be found in several adult tissues [126]. MSCs, isolated from bone marrow, are able to differentiate into specialized cells from mesoderm. These cells are interesting for regenerative medicine and the treatment of chronic diseases because of their good differentiation ability, immunogenicity, homing ability, banking and cryopreservation. Research is still needed, however, on how to distinguish MSCs in mixed-population cells [127]. The International Society for Cellular Therapy (ISCT) and The International Federation for Adipose Therapeutics (IFATS) have set out fundamental criteria for MSCs: plastic adherence, a specific set of cell surface markers (CD73, CD90, CD73, CD105) and lack of expression of CD14, CD34, CD45 and human leucocyte antigen-DR (HLA-DR) surface proteins, and the ability to differentiate into adipocyte, chondroblasts and osteoblast in vitro [124].

Human MSCs can be isolated not only from bone marrow but also from many other tissues [126], e.g., adipose tissue, amniotic fluid, amniotic membrane, dental tissue, endometrium, limb bud, menstrual blood, peripheral blood, placenta and fetal membrane, salivary gland, skin, foreskin, sub-amniotic umbilical cord lining membrane, synovial fluid and Wharton’s jelly [127]. Except for bone marrow MSCs, non-invasive methods are used for obtaining MSCs from other tissues, and a method for their isolation, characterization and expansion is crucial for their usage in regenerative medicine.

During long-term in vitro culturing of MSCs, their differentiation potential declines and the telomere length is reduced, but the probability of malignant transformation increases. Serum and growth factors determine MSCs’ properties during long-term in vitro culturing [126]. Using sufficient media and growth factors, MSCs are capable of differentiation into all three lineages—ectoderm, mesoderm and endoderm [127].

## 7. Current Practice and Future Directions

Many commercially available conventional materials are used in regenerative dentistry. Brief listing of materials for regenerative periodontal therapy is stated in Table 7 [128].

Recently, graphene and its derivatives, such as graphene oxide and reduced graphene oxide, have appeared as a very promising material for procedures of repair and regeneration of tissues, improving the mechanical strength of the polymeric scaffolds. Graphene is a synthetic single layer of aromatic carbon atoms with sp^2^ bonds. It has been stated that graphene-based materials regulate cell behavior, help in differentiation, and improve adhesion, growth and proliferation of cells. Despite the advantages of graphene-based materials, their toxicity at higher concentrations and their non-biodegradable nature needs further investigation. Further comparative studies between graphene-based materials and polymers should be completed before commercializing them in the market [130].

Recently, research in the dental and maxillofacial fields have paid attention to using oral-derived MSCs. In view of the fact that dental tissues are rich in MSCs, and thanks to easy access to the oral cavity, oral-derived MSCs could be a promising tool in dental and maxillofacial applications. However, it is necessary to explore the biology of these cells to transfer scientific results from laboratories to patients [131].

## 8. Conclusions

The aim for the future is to introduce existing processes and technologies into the clinical area to enhance patient care. An example of a future focus for intraoral tissue engineering is a treatment of bone degenerative processes to prepare conditions for fixation of dental implants. There is a tendency to optimize oral tissue engineering therapies directly for individual patients and combine approaches to make grow again of damaged tissue predictably.

Bone tissue engineering is a rapidly developing branch, and researchers are focusing on developing and improving new materials to imitate the biological environment of the body as authentically as possible [10]. Because a bone abounds in mechanical strength due to mineral components, a composite construct, a scaffold (of polymer with HAp and tricalcium phosphate) creates a natural environment for cell adhesion, osteogenic differentiation, and tissue formation. These composite materials play an important role in the regenerative processes of tissue. 

There have been many promising results in vitro, but unfortunately, only a few of them were successful in vivo [132]. A considerable improvement in scaffold structures was brought about by the introduction of 3D printing and its application to cells and biologics [10]. In the future bioprinting technologies seem very promising for a very wide range of applications in regenerative and transplantation medicine, dental implant treatment, drug screening, and cancer research [133]. There is a trend in developing biomaterials for injectable applications to avoid, as much as possible, invasive surgery and to develop techniques to generate nanofibrous scaffolds [11,134]. There should be a continuing of oral structure extension (such as alveolar bone, soft tissues of the teeth, and dental implants), especially using less invasive technologies to promote and accelerate tissue repair and regeneration.

## Figures and Tables

**Table 1:** Bone graft activity by type.

Graft	Osteogenesis	Osteoconduction	Osteoinduction	Mechanical Properties	Vascularity
Autograft					
Bone marrow	++	+/−	+	−	−
Cancellous	++	++	+	+	−
Cortical	+	+	+/−	++	−
Vascularized	++	++	+	++	++
Allograft					
Cancellous	−	++	+	+	−
Cortical	−	+/−	+/−	++	−
Demineralized	−	++	+++	−	−

+, ++, +++ = extent of activity: − = no activity, +++ = maximal activity.

**Table 2:** Classification of hydrogels based on different aspects.

Cross-Linking		Physical State	Source	Preparation	Degradation
Chemically	Physically (Self-Assembled)				
chemical cross-linking	freeze thawing	solid	natural	copolymeric	biodegradable
grafting—chemical, radiation	stereocomplex formation	semi- solid			
radical polymeration	ionic interaction		synthetic	homopolymeric	non-biodegradable
condensation	h-bonding	liquid			
enzymatic polymeration	maturation (heat-induced aggregation)		hybrid	interpenetrating	
high energy radiation					

**Table 3:** Types of materials used in tissue engineering and their advantages and disadvantages.

Type of material	Advantages	Disadvantages
Metals	Biocompatibility, non-toxicity and corrosion resistance	Not biodegradable
Bioceramics		
Bioactive glasses	Improve differentiation and osteogenesis	Low strength and brittleness
Hydroxyapatite	Bioactivity, biocompatibility, osteoconductivity, non-toxicity and non-inflammatory	Brittle, very slow degradation
Tricalcium phosphate	Supports in vivo osteogenic differentiation	Slow degradation, incompressible nature
Natural polymers		
Collagen	Enzymatic biodegradability	Complexity of structure
Gelatin	Biocompatible, biodegradable	Poor mechanical properties
Chitosan	Support cell attachment, differentiation, and migration, non-toxicity, non-allergenicity, mucoadhesivity, biocompatibility, biodegradability and osteoconductivity	Poor mechanical strength
Hyaluronic acid	Biocompatibility, biodegradability, viscoelasticity, enzymatic biodegradability	Very rapid degradation and water solubility
Alginate	Biocompatibility, easy gelling, easy chemical modification	Non-degradable in mammals, sterilization causes degradation
Agarose	Wide range of gelling and melting temperatures, no need cross-linking agents, little inflammatory response in vivo	Poor cell attachment
Synthetic polymers		
Poly(α-hydroxy acids)	Degradation products can be excluded from the body	Degradation by bulk erosion, relatively poor mechanical properties, hydrophobicity of the polymer surface
Poly(ε-caprolactone)	Biodegradable, non-toxic, a low melting point	Hydrophobicity, slow degradation
Polyurethanes	Excellent mechanical properties, good biocompatibility	Toxicity of degradation products (from aromatic diisocyanate component)

**Table 4:** Advantages and disadvantages of different SFFT types.

Techniques	Materials	Advantages	Disadvantages
Stereolithography (SL)	PEG, PEGDA, PPF, PCL, PDLLA	High accuracy, complex 3D structure including agents and cells, easy removal of photopolymer by heating	Photo-polymerization of materials, photocurable materials, expensive materials and equipment
Fused deposition modeling (FDM)	Thermoplastic polymers and their composites (PVA, ABSP400)	High porosity, complete pore interconnectivity, possibility of controlling porosity and size of pores, macro shape control, good compressive strength, solvent-free	High processing temperature, limited material range, inconsistency in pores,
Selective laser sintering (SLS)	Polymer ceramics (PCL, HAp, TCP)	Complex structure, possibility of controlling porosity and size of pores independently, wide range of powder materials, solvent-free, any secondary binder system	High processing temperature, using only thermally stable polymers, limited to small pore size
3D printing (3D-P)	Ceramics, polymers, metals	Easy process, high porosity, complete pore interconnectivity, possibility of controlling porosity and size of pores independently, macro shape control, wide range of materials	Use of toxic organic solvent, lack of mechanical strength, limited to small pore size

PEG: polyethylenglycol, PEGDA: poly(ethylene glycol)diacrylate, PPF: polypropylene fumarate, PVA: polyvinyl alcohol, ABSP400: acrylonitrile-butadiene-styrene, PCL: polycaprolactone, PDLLA: poly *D*,*L*-lactide, HAp: hydroxyapatite, TCP: tricalcium phosphate.

**Table 5:** Manufacturing technologies of scaffolds.

Type of Technology	Advantages	Disadvantages
Solvent casting and particulate leaching	Simple production, easy method, pore size and porosity can be controlled	Remaining toxic solvent can cause denaturation of incorporated molecules, a decrease in the activity of bioinductive molecules, impossibility of adding pharmacological agents
Gass foaming	Any organic solvents, carbon dioxide as a porogen gas, low toxic and non-flammable, simple and financially viable method	Cannot be used for hydrophilic and glassy polymers (low solubility in CO_2_)
Thermally induce phase separation (TIPS)	Porous polymer membrane of anisotropic and tubular 3D scaffolds, a low probability of defects	Not very suitable for seeding of the osteoblasts or for bone-tissue growth - pore size (10-100µm), use of organic solvents
Solid free form fabrication (SFFT)	A complex, highly accurate, three-dimensional product	Toxic solvents, use of porogens, shape limitation
Microsphere sintering	Excellent mechanical properties of scaffolds	Higher temperature and a longer time, a smaller pore size with lower porosity
Emulsion freeze drying	Highly porous scaffolds	
Electrospinning	Large surface areas, superior mechanical properties, large scale productions, very thin fibers	Inadequate control of scaffold architecture, pore network and size, and suboptimal 3D scaffolds
3D bioprinting	Adequate size of well-interconnected pores	Lack of mechanical strength and integrity

**Table 6:** List of companies and commercial bioreactor systems.

Company	Product Description
Aastrom	System for stem cell expansion
Histogenics	NeoCart^®^ autologous engineered neocartilage, which utilizes bioreactor system
New Brunswick	System for scale-up of mammalian cells
Minucell and Minutissue	Various bioreactors for 3D tissue culture with gradient container, container tissue factory, and perfusion culture container
Synthecon	Many systems incl. the NASA-developed Rotating Cell Culture System and a Perfused Culture System
Pluristem Therapeutics	Patented PluriX™ 3D Bioreactor for expansion of marrow stromal cells
FiberCell™ Systems Inc.	Manufacture Hollow Bioreactors for endothelial and other mammalian cell culture
Biovest International	Autovax ID™ automated system for mammalian cell culture
Wyle Labs and Celdyne	Hydrodynamic focusing bioreactors, developed by NASA for cell expansion and culture

**Table 7:** Materials used for conventional periodontal regenerative therapy [128] and as growth-factor-based grafts for dental surgical procedures [129].

Commercial Name of the Product	Type of Material	Company
BioMed^®^; Calcitek	Resorbable collagen	Colla-Tec Inc., USA
PLGA: GC membrane	Poly lactic-co-glycolic acid	GC Corporation, Japan
ePTFE: GORE-TEX Regenerative Membrane^®^	Polytetrafluorethylene	W.L. Gore & Associates, Inc., USA
Jeil Ti mesh	Titanium	ProSeed, Japan
Emdogain^®^	Enamel matrix derivative (EMD) product	Biora AB, Sweden
HA: NEOBONE^®^	Hydroxyapatite	Covalent Materials, Japan
β-TCP: OSferion^®^	Tricalcium phosphate	Olympus, Japan
HA+β-TCP: Triosite™	Biphasic calcium phosphate	Zimmer, France
BBM: Bio-Oss^®^	Bovine bone mineral	Geistlich Biomaterials, Switzerland
GEM 21S^®^	Human Platelet-derived growth factor and β-TCP	Osteohealth, USA
Mucograft	Collagen	Geistlich Pharma North America Inc., USA
Matriderm^®^	Collagen–elastin	MedSkin Solution Dr. Suwelack AG, Germany

## References

[1] Berthiaume F., Maguire T.J., Yarmush M.L. (2011). Tissue Engineering and Regenerative Medicine: History, Progress, and Challenges. Annu. Rev. Chem. Biomol. Eng..

[2] Chaudhari A., Vig K., Baganizi D., Sahu R., Dixit S., Dennis V., Singh S., Pillai S. (2016). Future Prospects for Scaffolding Methods and Biomaterials in Skin Tissue Engineering: A Review. Int. J. Mol. Sci..

[3] O’brien F.J. (2011). Biomaterials & scaffolds for tissue engineering. Mater. Today.

[4] Dlaska C.E., Andersson G., Brittberg M., Suedkamp N.P., Raschke M.J., Schuetz M.A. (2015). Clinical translation in tissue engineering—The surgeon’s view. Curr. Mol. Biol. Rep..

[5] Langer R., Vacanti J.P. (1993). Tissue engineering. Science.

[6] Stratton S., Shelke N.B., Hoshino K., Rudraiah S., Kumbar S.G. (2016). Bioactive polymeric scaffolds for tissue engineering. Bioact. Mater..

[7] Yu J., Xia H., Ni Q.Q. (2018). A three-dimensional porous hydroxyapatite nanocomposite scaffold with shape memory effect for bone tissue engineering. J. Mater. Sci..

[8] Dhandayuthapani B., Yoshida Y., Maekawa T., Kumar D.S. (2011). polymeric scaffolds in tissue engineering application: A review. Int. J. Polym. Sci..

[9] Kattimani V.S., Kondaka S., Lingamaneni K.P. (2016). Hydroxyapatite–-Past, present, and future in bone regeneration. Bone Tissue Regen. Insights.

[10] Stevens M.M. (2008). Biomaterials for bone tissue engineering. Mater. Today.

[11] Rahman S., Nagrath M., Ponnusamy S., Arany P. (2018). Nanoscale and macroscale scaffolds with controlled-release polymeric systems for dental craniomaxillofacial tissue engineering. Materials.

[12] Huang G.T.-J. (2011). Dental pulp and dentin tissue engineering and regeneration–advancement and challenge. Front. Biosci..

[13] Bakhtiar H., Mazidi S A., Mohammadi Asl S., Ellini M.R., Moshiri A., Nekoofar M.H., Dummer P.M.H. (2018). The role of stem cell therapy in regeneration of dentine-pulp complex: A systematic review. Prog. Biomater..

[14] Kaneko T., Gu B., Sone P.P., Zaw S.Y.M., Murano H., Zaw Z.C.T., Okiji T. (2018). Dental pulp tissue engineering using mesenchymal stem cells: A review with a protocol. Stem Cell Rev. Rep..

[15] Bottino M.C., Pankajakshan D., Nör J.E. (2017). Advanced scaffolds for dental pulp and periodontal regeneration. Dent. Clin. North Am..

[16] Pilipchuk S.P., Plonka A.B., Monje A., Taut A.D., Lanis A., Kang B., Giannobile W.V. (2015). Tissue engineering for bone regeneration and osseointegration in the oral cavity. Dent. Mater..

[17] Reena R., Nico H., Dieter W. (2015). Current concepts of bone regeneration in implant dentistry. J. Surg..

[18] Wang X., Xu S., Zhou S., Xu W., Leary M., Choong P., Qian M., Brandt M., Xie Y.M. (2016). Topological design and additive manufacturing of porous metals for bone scaffolds and orthopaedic implants: A review. Biomaterials.

[19] Rho J.Y., Kuhn-Spearing L., Zioupos P. (1998). Mechanical properties and the hierarchical structure of bone. Med. Eng. Phys..

[20] Bose S., Vahabzadeh S., Bandyopadhyay A. (2013). Bone tissue engineering using 3D printing. Mater. Today.

[21] Alford A.I., Kozloff K.M., Hankenson K.D. (2015). Extracellular matrix networks in bone remodeling. Int. J. Biochem. Cell Biol..

[22] Wang W., Yeung K.W.K. (2017). Bone grafts and biomaterials substitutes for bone defect repair: A review. Bioact. Mater..

[23] Roberts T.T., Rosenbaum A.J. (2012). Bone grafts, bone substitutes and orthobiologics: The bridge between basic science and clinical advancements in fracture healing. Organogenesis.

[24] Khan S.N., Cammisa F.P., Sandhu H.S., Diwan A.D., Girardi F.P., Lane J.M. (2005). The biology of bone grafting. J. Am. Acad. Orthop. Surg..

[25] Albrektsson T., Johansson C. (2001). Osteoinduction, osteoconduction and osseointegration. Eur. Spine J..

[26] Ge Z., Jin Z., Cao T. (2008). Manufacture of degradable polymeric scaffolds for bone regeneration. Biomed. Mater..

[27] Chen Q.Z., Thompson I.D., Boccaccini A.R. (2006). 45S5 Bioglass®-derived glass–Ceramic scaffolds for bone tissue engineering. Biomaterials.

[28] Varaprasad K., Raghavendra G.M., Jayaramudu T., Yallapu M.M., Sadiku R. (2017). A mini review on hydrogels classification and recent developments in miscellaneous applications. Mater. Sci. Eng. C.

[29] Ullah F., Othman M.B.H., Javed F., Ahmad Z., Akil H.M. (2015). Classification, processing and application of hydrogels: A review. Mater. Sci. Eng. C.

[30] Tan H., Chu C.R., Payne K.A., Marra K.G. (2009). Injectable in situ forming biodegradable chitosan–hyaluronic acid based hydrogels for cartilage tissue engineering. Biomaterials.

[31] Hoffman A.S. (2012). Hydrogels for biomedical applications. Adv. Drug Deliv. Rev..

[32] Hench L.L., Polak J.M. (2002). Third-generation biomedical materials. Science.

[33] Gunatillake P.A., Adhikari R. (2003). Biodegradable synthetic polymers for tissue engineering. Eur. Cells Mater..

[34] Rezwan K., Chen Q.Z., Blaker J.J., Boccaccini A.R. (2006). Biodegradable and bioactive porous polymer/inorganic composite scaffolds for bone tissue engineering. Biomaterials.

[35] Dorati R., DeTrizio A., Modena T., Conti B., Benazzo F., Gastaldi G., Genta I. (2017). Biodegradable scaffolds for bone regeneration combined with drug-delivery systems in osteomyelitis therapy. Pharmaceuticals.

[36] Kokubo T., Takadama H. (2006). How useful is SBF in predicting in vivo bone bioactivity?. Biomaterials.

[37] Sasikumar S., Ravy L. (2015). Influence of needle-like morphology on the bioactivity of nanocrystalline wollastonite–an in vitro study. Int. J. Nanomed..

[38] Gotman I. (1997). Characteristics of Metals Used in Implants. J. Endourol..

[39] Babuska V., Moztarzadeh O., Kubikova T., Moztarzadeh A., Hrusak D., Tonar Z. (2016). Evaluating the osseointegration of nanostructured titanium implants in animal models: Current experimental methods and perspectives (Review). Biointerphases.

[40] AzoMaterials. https://www.azom.com/article.aspx?ArticleID=14935.

[41] Babuska V., Dobra J., Kulda V., Kripnerova M., Moztarzadeh A., Bolek L., Lahoda J., Hrusak D. (2015). Comparison of fibroblast and osteoblast response to cultivation on titanium implants with different grain sizes. J. Nanomater..

[42] Turnbull G., Clarke J., Picard F., Riches P., Jia L., Han F., Li B., Shu W. (2018). 3D bioactive composite scaffolds for bone tissue engineering. Bioact. Mater..

[43] Liu J., Ruan J., Chang L., Yang H., Ruan W. (2017). Porous Nb-Ti-Ta alloy scaffolds for bone tissue engineering: Fabrication, mechanical properties and in vitro/vivo biocompatibility. Mater. Sci. Eng. C.

[44] Miguez-Pacheco V., Hench L.L., Boccaccini A.R. (2015). Bioactive glasses beyond bone and teeth: Emerging applications in contact with soft tissues. Acta Biomater..

[45] Jell G., Stevens M.M. (2006). Gene activation by bioactive glasses. J. Mater. Sci. Mater. Med..

[46] Hu J., Zhu Y., Tong H., Shen X., Chen L., Ran J. (2016). A detailed study of homogeneous agarose/hydroxyapatite nanocomposites for load-bearing bone tissue. Int. J. Biol. Macromol..

[47] Asti A., Gioglio L. (2014). Natural and synthetic biodegradable polymers: Different scaffolds for cell expansion and tissue formation. Int. J. Artif. Organs.

[48] Kartikasari N., Yuliati A., Listiana I., Setijanto D., Suardita K., Ariani D., Sosiawan A. Characteristic of bovine hydroxyapatite-gelatin-chitosan scaffolds as biomaterial candidate for bone tissue engineering. Proceedings of the 2016 IEEE EMBS Conference on Biomedical Engineering and Sciences (IECBES).

[49] Gentile P., Nandagiri V.K., Daly J., Chiono V., Mattu C., Tonda-Turo C., Ciardelli G., Ramtoola Z. (2016). Localised controlled release of simvastatin from porous chitosan–gelatin scaffolds engrafted with simvastatin loaded PLGA-microparticles for bone tissue engineering application. Mater. Sci. Eng. C.

[50] Lien S.-M., Ko L.-Y., Huang T.-J. (2009). Effect of pore size on ECM secretion and cell growth in gelatin scaffold for articular cartilage tissue engineering. Acta Biomater..

[51] Wu X., Liu Y., Li X., Wen P., Zhang Y., Long Y., Wang X., Guo Y., Xing F., Gao J. (2010). Preparation of aligned porous gelatin scaffolds by unidirectional freeze-drying method. Acta Biomater..

[52] Saravanan S., Leena R.S., Selvamurugan N. (2016). Chitosan based biocomposite scaffolds for bone tissue engineering. Int. J. Biol. Macromol..

[53] Rajan Unnithan A., Ramachandra Kurup Sasikala A., Park C.H., Kim C.S. (2017). A unique scaffold for bone tissue engineering: An osteogenic combination of graphene oxide–hyaluronic acid–chitosan with simvastatin. J. Ind. Eng. Chem..

[54] LogithKumar R., KeshavNarayan A., Dhivya S., Chawla A., Saravanan S., Selvamurugan N. (2016). A review of chitosan and its derivatives in bone tissue engineering. Carbohydr. Polym..

[55] Balagangadharan K., Dhivya S., Selvamurugan N. (2017). Chitosan based nanofibers in bone tissue engineering. Int. J. Biol. Macromol..

[56] Di Martino A., Sittinger M., Risbud M.V. (2005). Chitosan: A versatile biopolymer for orthopaedic tissue-engineering. Biomaterials.

[57] Swetha M., Sahithi K., Moorthi A., Srinivasan N., Ramasamy K., Selvamurugan N. (2010). Biocomposites containing natural polymers and hydroxyapatite for bone tissue engineering. Int. J. Biol. Macromol..

[58] Deng Y., Ren J., Chen G., Li G., Wu X., Wang G., Gu G., Li J. (2017). Injectable in situ cross-linking chitosan-hyaluronic acid based hydrogels for abdominal tissue regeneration. Sci. Rep..

[59] Gwon K., Kim E., Tae G. (2017). Heparin-hyaluronic acid hydrogel in support of cellular activities of 3D encapsulated adipose derived stem cells. Acta Biomater..

[60] Nimmo C.M., Owen S.C., Shoichet M.S. (2011). Diels−Alder click cross-linked hyaluronic acid hydrogels for tissue engineering. Biomacromolecules.

[61] Collins M.N., Birkinshaw C. (2013). Hyaluronic acid based scaffolds for tissue engineering—A review. Carbohydr. Polym..

[62] Lee K.Y., Mooney D.J. (2012). Alginate: Properties and biomedical applications. Prog. Polym. Sci..

[63] Pawar S.N., Edgar K.J. (2012). Alginate derivatization: A review of chemistry, properties and applications. Biomaterials.

[64] Gómez-Mascaraque L.G., Méndez J.A., Fernández-Gutiérrez M., Vázquez B., San Román J. (2014). Oxidized dextrins as alternative crosslinking agents for polysaccharides: Application to hydrogels of agarose–chitosan. Acta Biomater..

[65] Zarrintaj P., Manouchehri S., Ahmadi Z., Saeb M.R., Urbanska A.M., Kaplan D.L., Mozafari M. (2018). Agarose-based biomaterials for tissue engineering. Carbohydr. Polym..

[66] Marras-Marquez T., Peña J., Veiga-Ochoa M.D. (2014). Agarose drug delivery systems upgraded by surfactants inclusion: Critical role of the pore architecture. Carbohydr. Polym..

[67] Barrangou L.M., Daubert C.R., Allen Foegeding E. (2006). Textural properties of agarose gels. I. Rheological and fracture properties. Food Hydrocolloids.

[68] Zhang L.-M., Wu C.-X., Huang J.-Y., Peng X.-H., Chen P., Tang S.-Q. (2012). Synthesis and characterization of a degradable composite agarose/HA hydrogel. Carbohydr. Polym..

[69] Khanarian N.T., Haney N.M., Burga R.A., Lu H.H. (2012). A functional agarose-hydroxyapatite scaffold for osteochondral interface regeneration. Biomaterials.

[70] Watanabe J., Kashii M., Hirao M., Oka K., Sugamoto K., Yoshikawa H., Akashi M. (2007). Quick-forming hydroxyapatite/agarose gel composites induce bone regeneration. J. Biomed. Mater. Res. A.

[71] Zhang R., Ma P.X. (1999). Poly (α-hydroxyl acids)/hydroxyapatite porous composites for bone-tissue engineering. I. Preparation and morphology. J. Biomed. Mater..

[72] Yu N.Y.C., Schindeler A., Little D.G., Ruys A.J. (2010). Biodegradable poly(α-hydroxy acid) polymer scaffolds for bone tissue engineering. J. Biomed. Mater. Res. B Appl. Biomater..

[73] Grossen P., Witzigmann D., Sieber S., Huwyler J. (2017). PEG-PCL-based nanomedicines: A biodegradable drug delivery system and its application. J. Control. Release.

[74] Dash T.K., Konkimalla V.B. (2012). Poly-epsilon-caprolactone based formulations for drug delivery and tissue engineering: A review. J. Control. Release.

[75] Wu F., Liu C., O’Neill B., Wei J., Ngothai Y. (2012). Fabrication and properties of porous scaffold of magnesium phosphate/polycaprolactone biocomposite for bone tissue engineering. Appl. Surf. Sci..

[76] Williams J.M., Adewunmi A., Schek R.M., Flanagan C.L., Krebsbach P.H., Feinberg S.E., Hollister S.J., Das S. (2005). Bone tissue engineering using polycaprolactone scaffolds fabricated via selective laser sintering. Biomaterials.

[77] Gabriel L.P., dos Santos M.E.M., Jardini A.L., Bastos G.N.T., Dias C.G.B.T., Webster T.J., Maciel Filho R. (2017). Bio-based polyurethane for tissue engineering applications: How hydroxyapatite nanoparticles influence the structure, thermal and biological behavior of polyurethane composites. Nanomed. Nanotechnol. Biol. Med..

[78] Ryszkowska J.L., Auguścik M., Sheikh A., Boccaccini A.R. (2010). Biodegradable polyurethane composite scaffolds containing Bioglass^®^ for bone tissue engineering. Compos. Sci. Technol..

[79] Vacanti J.P., Langer R. (1999). Tissue engineering: The design and fabrication of living replacement devices for surgical reconstruction and transplantation. Lancet.

[80] Jiang W., Cipriano A.F., Tian Q., Zhang C., Lopez M., Sallee A., Lin A., Cortez Alcaraz M.C., Wu Y., Zheng Y. (2018). In vitro evaluation of MgSr and MgCaSr alloys via direct culture with bone marrow derived mesenchymal stem cells. Acta Biomater..

[81] Liu X., Ma P.X. (2004). Polymeric scaffolds for bone tissue engineering. Ann. Biomed. Eng..

[82] Mikos A.G., Temenoff J.S. (2000). Formation of highly porous biodegradable scaffolds for tissue engineering. Electron. J. Biotechnol..

[83] Suh S.W., Shin J.Y., Kim J., Kim J., Beak C.H., Kim D.-I., Kim H., Jeon S.S., Choo I.-W. (2002). Effect of different particles on cell proliferation in polymer scaffolds using a solvent-casting and particulate leaching technique. ASAIO J..

[84] Ji C., Annabi N., Khademhosseini A., Dehghani F. (2011). Fabrication of porous chitosan scaffolds for soft tissue engineering using dense gas CO_2_. Acta Biomater..

[85] Nam Y.S., Yoon J.J., Park T.G. (2000). A novel fabrication method of macroporous biodegradable polymer scaffolds using gas foaming salt as a porogen additive. J. Biomed. Mater. Res..

[86] Liang H.Q., Wu Q.Y., Wan L.S., Huang X.J., Xu Z.K. (2013). Polar polymer membranes via thermally induced phase separation using a universal crystallizable diluent. J. Membr. Sci..

[87] Jung J.T., Kim J.F., Wang H.H., di Nicolo E., Drioli E., Lee Y.M. (2016). Understanding the non-solvent induced phase separation (NIPS) effect during the fabrication of microporous PVDF membranes via thermally induced phase separation (TIPS). J. Membr. Sci..

[88] Yuan B., Zhou S., Chen X. (2017). Rapid prototyping technology and its application in bone tissue engineering. J. Zhejiang Univ.-Sci. B.

[89] Leong K.F., Cheah C.M., Chua C.K. (2003). Solid freeform fabrication of three-dimensional scaffolds for engineering replacement tissues and organs. Biomaterials.

[90] Lee J.W., Kim J.Y., Cho D.-W. (2010). Solid free-form fabrication technology and its application to bone tissue engineering. Int. J. Stem Cells.

[91] Stevens B., Yang Y., Mohandas A., Stucker B., Nguyen K.T. (2008). A review of materials, fabrication methods, and strategies used to enhance bone regeneration in engineered bone tissues. J. Biomed. Mater. Res. B Appl. Biomater..

[92] Shi X., Su K., Varshney R.R., Wang Y., Wang D.-A. (2011). Sintered microsphere scaffolds for controlled release and tissue engineering. Pharm. Res..

[93] Wang Y., Shi X., Ren L., Wang C., Wang D.-A. (2009). Porous poly (lactic-co-glycolide) microsphere sintered scaffolds for tissue repair applications. Mater. Sci. Eng. C.

[94] Jeon J.H., Bhamidipati M., Sridharan B., Scurto A.M., Berkland C.J., Detamore M.S. (2013). Tailoring of processing parameters for sintering microsphere-based scaffolds with dense-phase carbon dioxide. J. Biomed. Mater. Res. B Appl. Biomater..

[95] Alizadeh M., Abbasi F., Khoshfetrat A.B., Ghaleh H. (2013). Microstructure and characteristic properties of gelatin/chitosan scaffold prepared by a combined freeze-drying/leaching method. Mater. Sci. Eng. C Mater. Biol. Appl..

[96] Fu S.Z., Wang X.H., Guo G., Shi S.A., Liang H., Luo F., Wei Y.Q., Qian Z.Y. (2010). Preparation and characterization of nano-hydroxyapatite/poly(epsilon-caprolactone)-poly(ethylene glycol)-poly(epsilon-caprolactone) composite fibers for tissue engineering. J. Phys. Chem. C.

[97] Agarwal S., Wendorff J.H., Greiner A. (2008). Use of electrospinning technique for biomedical applications. Polymer.

[98] Venugopal J., Vadgama P., Kumar T.S.S., Ramakrishna S. (2007). Biocomposite nanofibres and osteoblasts for bone tissue engineering. Nanotechnology.

[99] An J., Teoh J.E.M., Suntornnond R., Chua C.K. (2015). Design and 3D printing of scaffolds and tissues. Engineering.

[100] Gungor-Ozkerim P.S., Inci I., Zhang Y.S., Khademhosseini A., Dokmeci M.R. (2018). Bioinks for 3D bioprinting: An overview. Biomater. Sci..

[101] Hospodiuk M., Dey M., Sosnoski D., Ozbolat I.T. (2017). The bioink: A comprehensive review on bioprintable materials. Biotechnol. Adv..

[102] Wen Y., Xun S., Haoye M., Baichuan S., Peng C., Xuejian L., Kaihong Z., Xuan Y., Jiang P., Shibi L. (2017). 3D printed porous ceramic scaffolds for bone tissue engineering: A review. Biomater. Sci..

[103] Shaunak S., S Dhinsa B., S Khan W. (2017). The role of 3D modelling and printing in orthopaedic tissue engineering: A review of the current literature. Curr. Stem Cell Res. Ther..

[104] Zhu W., Ma X., Gou M., Mei D., Zhang K., Chen S. (2016). 3D printing of functional biomaterials for tissue engineering. Curr. Opin. Biotechnol..

[105] Mandrycky C., Wang Z., Kim K., Kim D.-H. (2016). 3D bioprinting for engineering complex tissues. Biotechnol. Adv..

[106] Jia W., Gungor-Ozkerim P.S., Zhang Y.S., Yue K., Zhu K., Liu W., Pi Q., Byambaa B., Dokmeci M.R., Shin S.R. (2016). Direct 3D bioprinting of perfusable vascular constructs using a blend bioink. Biomaterials.

[107] Zhu W., Qu X., Zhu J., Ma X., Patel S., Liu J., Wang P., Lai C.S.E., Gou M., Xu Y. (2017). Direct 3D bioprinting of prevascularized tissue constructs with complex microarchitecture. Biomaterials.

[108] Sun J., Vijayavenkataraman S., Liu H. (2017). An overview of scaffold design and fabrication technology for engineered knee meniscus. Materials.

[109] Vijayavenkataraman S., Zhang S., Lu W.F., Fuh J.Y.H. (2018). Electrohydrodynamic-jetting (EHD-jet) 3D-printed functionally graded scaffolds for tissue engineering applications. J. Mater. Res..

[110] Tang D., Tare R.S., Yang L.-Y., Williams D.F., Ou K.-L., Oreffo R.O.C. (2016). Biofabrication of bone tissue: Approaches, challenges and translation for bone regeneration. Biomaterials.

[111] Grayson W.L., Bhumiratana S., Cannizzaro C., Chao P.-H.G., Lennon D.P., Caplan A.I., Vunjak-Novakovic G. (2008). Effects of initial seeding density and fluid perfusion rate on formation of tissue-engineered bone. Tissue Eng. Part A.

[112] Egger D., Spitz S., Fischer M., Handschuh S., Glösmann M., Friemert B., Egerbacher M., Kasper C. (2017). Application of a parallelizable perfusion bioreactor for physiologic 3D cell culture. Cells Tissues Organs.

[113] Martin I., Wendt D., Heberer M. (2004). The role of bioreactors in tissue engineering. Trends Biotechnol..

[114] Zhao J., Griffin M., Cai J., Li S., Bulter P.E.M., Kalaskar D.M. (2016). Bioreactors for tissue engineering: An update. Biochem. Eng. J..

[115] Beşkardeş I.G., Aydın G., Bektaş Ş., Cengiz A., Gümüşderelioğlu M. (2018). A systematic study for optimal cell seeding and culture conditions in a perfusion mode bone-tissue bioreactor. Biochem. Eng. J..

[116] Stiehler M., Bünger C., Baatrup A., Lind M., Kassem M., Mygind T. (2008). Effect of dynamic 3-D culture on proliferation, distribution, and osteogenic differentiation of human mesenchymal stem cells. J. Biomed. Mater. Res. Part A.

[117] Mygind T., Stiehler M., Baatrup A., Li H., Zou X., Flyvbjerg A., Kassem M., Bünger C. (2007). Mesenchymal stem cell ingrowth and differentiation on coralline hydroxyapatite scaffolds. Biomaterials.

[118] Sikavitsas V.I., Bancroft G.N., Mikos A.G. (2002). Formation of three-dimensional cell/polymer constructs for bone tissue engineering in a spinner flask and a rotating wall vessel bioreactor. J. Biomed. Mater. Res. Part A.

[119] Goldstein A.S., Juarez T.M., Helmke C.D., Gustin M.C., Mikos A.G. (2001). Effect of convection on osteoblastic cell growth and function in biodegradable polymer foam scaffolds. Biomaterials.

[120] Yeatts A.B., Fisher J.P. (2011). Bone tissue engineering bioreactors: Dynamic culture and the influence of shear stress. Bone.

[121] Ciuffi S., Zonefrati R., Brandi M.L. (2017). Adipose stem cells for bone tissue repair. Clin. Cases Miner. Bone Metab..

[122] Gimble J.M., Katz A.J., Bunnell B.A. (2007). Adipose-derived stem cells for regenerative medicine. Circ. Res..

[123] Gao S., Zhao P., Lin C., Sun Y., Wang Y., Zhou Z., Yang D., Wang X., Xu H., Zhou F. (2014). Differentiation of human adipose-derived stem cells into neuron-like cells which are compatible with photocurable three-dimensional scaffolds. Tissue Eng. Part A.

[124] Skubis A., Sikora B., Zmarzły N., Wojdas E., Mazurek U. (2016). Adipose-derived stem cells: A review of osteogenesis differentiation. Folia Biol. Oecol..

[125] Bunnell B., Flaat M., Gagliardi C., Patel B., Ripoll C. (2008). Adipose-derived stem cells: Isolation, expansion and differentiation. Methods.

[126] Aggarwal S., Pittenger M.F. (2005). Human mesenchymal stem cells modulate allogeneic immune cell responses. Blood.

[127] Ullah I., Subbarao R.B., Rho G.J. (2015). Human mesenchymal stem cells - current trends and future prospective. Biosci. Rep..

[128] Egusa H., Sonoyama W., Nishimura M., Atsuta I., Akiyama K. (2012). Stem cells in dentistry—Part II: Clinical applications. J. Prosthodont. Res..

[129] Abou Neel E.A., Chrzanowski W., Salih V.M., Kim H.-W., Knowles J.C. (2014). Tissue engineering in dentistry. Dentistry J..

[130] Prasadh S., Suresh S., Wong R. (2018). Osteogenic potential of graphene in bone tissue engineering scaffolds. Materials.

[131] Spagnuolo G., Codispoti B., Marrelli M., Rengo C., Rengo S., Tatullo M. (2018). Commitment of oral-derived stem cells in dental and maxillofacial applications. Dentistry J..

[132] Holzwarth J.M., Ma P.X. (2011). Biomimetic nanofibrous scaffolds for bone tissue engineering. Biomaterials.

[133] Ozbolat I.T., Peng W., Ozbolat V. (2016). Application areas of 3D bioprinting. Drug Discov. Today.

[134] Jahangirian H., Ghasemian Lemraski E., Rafiee-Moghaddam R., Webster T. (2018). A review of using green chemistry methods for biomaterials in tissue engineering. Int. J. Nanomed..

